# Correlations between Ki67 expression and uptake of ^68^Ga-FAPI-04 versus ^18^F-FDG in different types of tumors: a lesion-based study

**DOI:** 10.1186/s40644-025-00974-x

**Published:** 2025-12-17

**Authors:** Jia Deng, Die You, Chunfang Zhang, Dengsai Peng, Jiao Ma, Yue Chen

**Affiliations:** 1 Department of Nuclear Medicine, Affiliated Hospital of Southwest Medical University, Luzhou, Sichuan 646000 China; 2 Nuclear Medicine and Theranostics Key Laboratory of Sichuan Province, Luzhou, Sichuan 646000 China; 3 Institute of Nuclear Medicine, Southwest Medical University, Luzhou, Sichuan 646000 China

**Keywords:** ^68^Ga-FAPI-04, ^18^F-FDG, Immunohistochemistry, Ki67

## Abstract

**Background:**

This study aimed to evaluate the correlation between Ki67 expression and uptake values of ^68^Ga-FAPI-04 and ^18^F-FDG PET/CT in different types of tumor lesions.

**Methods:**

Patients who underwent concurrent ^68^Ga-FAPI-04 and ^18^F-FDG PET/CT and were candidates for biopsy of any lesion positive on either imaging agent were enrolled in this retrospective study. Receiver operating characteristic (ROC) curve analysis was conducted on Ki67 prediction of ^18^F-FDG and ^68^Ga-FAPI-04 positive lesions. The maximum standard uptake values (SUVmax) and tumor-to-background ratio (TBR) of the lesions were collected. The mean SUVmax and mean TBR of lesions from different tumor groups were compared using the Kruskal-Wallis test. Spearman rho test was used to analyze the correlation between the same parameters of two types of PET. The protein expression of Ki67 was examined by immunohistochemical staining. The correlation between the parameters of two imaging agents and Ki67 expression in tissue samples was analyzed by Spearman’s rho test.

**Results:**

A total of 66 patients were included (31 men, 35 women; mean age 56.11 ± 12.64 years), with 69 malignant tumor samples analyzed. All tumors exhibited Ki67 expression, with varying intensity and positivity rates ranging from 1% to 95%. ROC curve analysis showed that Ki67 expression had a good ability to predict visual positive of ^18^F-FDG (AUC = 0.819, *p* < 0.0001), but it did not have statistical significance in predicting positive of ^68^Ga-FAPI-04 (AUC = 0.698, *p* = 0.069). The optimal critical value for Ki67 was 15%. Overall, ^18^F-FDG SUVmax and TBR were moderately positively correlated with Ki67 (rho = 0.471, *p* < 0.0001 and rho = 0.405, *P* < 0.001, respectively), while the ^68^Ga-FAPI-04 parameters showed no significant correlation with Ki67 (rho = 0.121, *p* = 0.321 and rho = 0.119, *P* = 0.329, respectively). In subgroup analysis, the correlation between ^18^F-FDG (SUVmax and TBR) and Ki67 remained moderate in lesions with Ki67 > 15% (rho = 0.346, *p* = 0.008 and rho = 0.327, *p* = 0.013, respectively). ^18^F-FDG (SUVmax and TBR) and Ki67 also showed a particularly correlation in tumor groups of hematology and head and neck tumors. In any tumor group or Ki67 subgroup, there was no significant association between ^68^Ga-FAPI-04 (SUVmax and TBR) and Ki67. Regarding the correlation between two imaging agents, the tracer correlation in Ki67≤15% lesions is stronger than that in Ki67 > 15% lesions. Unlike ^18^F-FDG, which showed no difference between tumor groups (SUVmax: *p* = 0.260, TBR: *p* = 0.072), significant heterogeneity in ^68^Ga-FAPI-04 uptake was observed between different tumor groups (SUVmax: *p* = 0.002, TBR: *p* = 0.004).

**Conclusion:**

Mechanistic differences likely exist between ^68^Ga-FAPI-04 and Ki67. In contrast, ^18^F-FDG may serve as a direct indicator for assessing tumor proliferative capacity. Limitations of this study could be addressed in future research with larger sample sizes and subgroup analyses to yield more valuable insights.

**Trial registration:**

ChiCTR, ChiCTR2100044131. Registered 10 October 2022, https://www.chictr.org.cn, ChiCTR2100044131.

## Introduction

Ki67 antigen, a nuclear protein existing as two isoforms (345 and 395 kDa), is a well-established marker of cell proliferation. Its expression is strictly confined to the active phases of the cell cycle (G1, S, G2, and M) and is absent in quiescent (G0) cells [1]. This profile makes it an attractive therapeutic target and powerful prognostic indicator for various malignant tumors [2–4]. However, reliance on biopsy for Ki67 assessment introduces significant limitations, including sampling bias due to tumor heterogeneity, procedural risks, and patient distress linked to repeated invasive procedures. Consequently, non-invasive imaging alternatives for profiling tumor proliferation are urgently needed.

Compared to traditional morphological imaging techniques, ^18^F-FDG PET/CT combines anatomical information and tumor metabolic activity. The standard uptake value (SUV), a semi-quantitative measure of glucose metabolic rate, can reflect tumor heterogeneity and has emerged as a potential predictor of treatment response [5, 6]. More recently, ^68^Ga-FAPI PET/CT has been developed to target fibroblast activation protein (FAP), visualizing the tumor stroma with high sensitivity, often surpassing that of ^18^F-FDG for detecting primary and metastatic lesions [7, 8]. This is particularly relevant given the growing recognition that cancer-associated fibroblasts (CAFs) are heterogeneous and play diverse roles in tumor growth, from modulating proliferation and metastasis to fostering immunosuppression and treatment resistance [9].

Establishing a reliable correlation between non-invasive PET parameters and the Ki67 proliferation index holds considerable clinical promise. Reliable non-invasive imaging methods could guide biopsies to the most aggressive tumor foci, improve risk stratification, and enable early monitoring of treatment efficacy by tracking changes in proliferative activity before anatomical shifts occur.

Despite the distinct biological mechanisms of ^18^F-FDG and ^68^Ga-FAPI-04, the relationship between their uptake and Ki67 expression remains ambiguous and appears to be cancer-type specific. For instance, while a positive correlation between Ki67 scores and SUVmax of ^18^F-FAPI-04 was reported in liver cancer [10], no significant association was found between the Ki67 and ^68^Ga-FAPI/^18^F-FDG PET parameters in gliomas [11]. This contradiction underscores the necessity for pan-cancer investigations to clarify the generalizability of these relationships. Therefore, this lesion-based study was designed to systematically evaluate and compare the correlations between Ki67 expression and the semi-quantitative parameters of ^18^F-FDG and ^68^Ga-FAPI-04 PET/CT across a wide range of tumor types.

## Materials and methods

### Study design and patient selection

We conducted a retrospective analysis of patients who underwent ^18^F-FDG PET/CT examinations and participated in the ^68^Ga-FAPI-04 PET/CT clinical trial in our department from March 2023 to December 2024 (approval no. KY2022114; clinical trial registration no. ChiCTR2200044131). Informed consent was obtained from all patients for both the ^68^Ga-FAPI PET/CT examination and their medical and imaging records. Additionally, we reviewed whether the patients underwent pathological examination of Ki67. If the malignant tumor site in the patient’s pathological biopsy showed positive uptake for either of the two imaging agents, the lesion was included in the analysis. To ensure a stable tumor biology for a direct comparison, the exclusion criteria were as follows: 1) Patients who underwent any anti-tumor therapy within four weeks prior to the first PET/CT scan; 2) Patients who received any anti-tumor treatment between the two PET/CT scans; 3) Ki67 biopsy was obtained beyond four weeks from any imaging time; 4) Patients for whom the time interval between the two PET/CT scans exceeded one week.

### Preparation of ^18^F-FDG and ^68^Ga-FAPI-04

^18^F-FDG was produced using a circuit-compatible ^18^F-FDG synthesis module following standard methods (FDG-N; PET Science & Technology, China). We purchased the precursor FAPI-04 from MCE (MedChemExpress, USA) with a purity grade of 98% and a mass of 872.91. The ^68^Ga-FAPI-04 labeling was carried out according to the method described previously [12, 13]. The radiochemical purity of ^68^Ga-FAPI-04 and ^18^F-FDG exceeded 95%. The final product is sterile and meets all of our institution’s required standards before use [13].

### PET imaging

For the acquisition of ^18^F-FDG PET/CT images, patients fasted for a minimum of 6 hours, and their plasma glucose levels were below 11 mmol/L. For ^68^Ga-FAPI-04 PET/CT examination, there was no requirement for any special preparation. Intravenous doses of ^18^F-FDG and ^68^Ga-FAPI-04 were 3.7 MBq/kg(0.1mCi/kg) and 1.85MBq/kg(0.05mCi/kg), respectively. Before scanning, the patient was informed to urinate and drink 500 milliliters of water to promote urinary excretion. Data were collected using a hybrid PET/CT scanner (uMI780, United Imaging Healthcare, Shanghai, China) 45 to 60 minutes after intravenous injection. CT imaging was conducted from the head down to the upper thigh segment, utilizing a tube current of 120 mA, a tube voltage of 120 kV, and a slice thickness of 3.00 mm. PET was then performed at the same bed position as the CT scan, with 5 to 6 bed positions in three-dimensional acquisition mode. ^18^F-FDG and ^68^Ga-FAPI-04 PET images were acquired in 1.5 and 3.0 minutes per position, respectively. ^68^Ga-FAPI-04 PET/CT was carried out within 7 days following ^18^F-FDG PET/CT. After the reconstruction was complete, image analysis was performed using the joint imaging post-processing fusion software. This part of the method drew reference from previous research [14].

### Image analysis

Two experienced nuclear medicine physicians interpreted the PET/CT images of both ^18^F-FDG and ^68^Ga-FAPI-04 in a randomized sequence. Any disagreements were settled through mutual agreement. Based on the normal biodistribution patterns of ^18^F-FDG and ^68^Ga-FAPI-04, we defined malignant lesions as focal areas of radiotracer uptake that were significantly higher than the surrounding background tissue and could not be attributed to physiological uptake or normal anatomical structures. Spherical regions of interest (VOIs) were employed to determine the maximum standardized uptake value (SUVmax) of the hottest lesion in each region and the tumor volume. The activity background was represented by the mean standardized uptake value (SUVmean) of a circular sphere with a diameter of 2 cm. It is particularly noteworthy that for hematological tumors, we have chosen the descending aorta as the background, which provides a stable blood pool reference standard. Whereas for non-hematological tumors, adipose tissue serves as the background, which is an area typically unaffected by the spread of solid tumors. The corresponding target-to-background ratio (TBR) was obtained by dividing the SUVmax of the lesion by the SUVmean of the defined background VOI.

### Immunohistochemistry and Ki67 staging

The Ki67 assessment was conducted on tumor specimens obtained during diagnostic biopsy or surgery of the patients. It was performed by pathologists from our hospital’s pathology department. Two pathologists conducted microscopic analysis of the slides. All stained nuclei were determined to be positive (regardless of intensity/distribution), and the average percentage was manually calculated. The final result was obtained through our medical record system.

### Statistical analysis

All statistical analyses were conducted using SPSS software (version 22.0; IBM, Armonk, NY). Continuous variables were presented as mean±standard deviation. Receiver operating characteristic (ROC) curve analysis was conducted on Ki67 prediction of ^18^F-FDG and ^68^Ga-FAPI-04 positive lesions. The normality of the SUVmax and TBR data distribution for each tumor group was assessed using the Shapiro-Wilk test. To compare the intergroup differences in SUVmax and TBR among different tumor groups, one-way ANOVA was used if the data were normally distributed; otherwise, the Kruskal-Wallis test was applied. Spearman’s rho correlation coefficient is used to evaluate the correlation between Ki67 and PET parameters, and this method was also used to evaluate the same parameter between the two PET. Correlation was considered low for 0 < r < 0.3, moderate for 0.3≤*r* < 0.5, strong for 0.5≤*r* < 0.8 and excellent for *r* ≥ 0.8. A *P*-value of < 0.05 was considered statistically significant.

## Results

### Patient characteristics

**Fig. 1:**
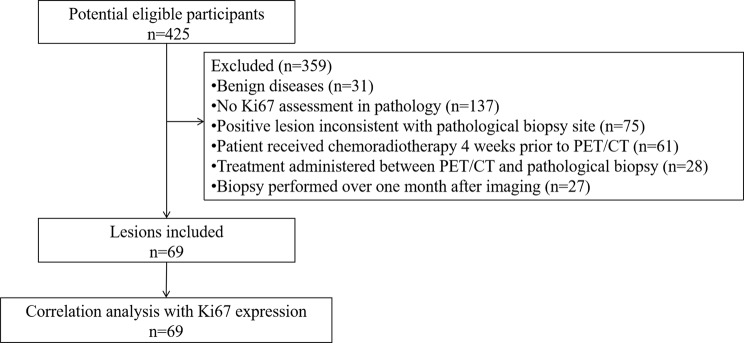
Flowchart of this study

Initially, 425 patients met the enrollment criteria for the clinical trial in our department from March 2023 to December 2024. After applying exclusion criteria of this retrospective study, 66 patients (69 lesions) were included. The study design is illustrated in Fig. 1. The study subjects comprised 31 men and 35 women with an average age of 56.11 ± 12.64 years (range, 19–81 years). There were no adverse events or complications observed in all patients who underwent ^18^F-FDG and ^68^Ga-FAPI-04 PET/CT examinations. Patient characteristics are summarized in Table 1.

**Table 1:** Summary of patient characteristic

Characteristic	Value
**No.Patient**	66
**Age, y**	
Mean±Standard deviation	56.11±12.64
**Sex**	
Male	31(47)
Female	35(53)
**No.Lesion**	69
**Lesion site**	
Digestive System	16(23.2)
Reproductive System	15(21.7)
Head and neck cancers	12(17.4)
Hematologic Tumors	11(15.9)
Respiratory System	6(8.7)
Mesenchymal origin	5(7.2)
Nervous System	2(2.9)
Thymic Tumor	2(2.9)

Note.—Data are presented as number of patients, with percentages in parentheses, unless stated otherwise

### Immunohistochemistry findings

Pathological biopsy was performed on 69 lesions (highly suspected of being malignant tumors), and Ki67 expression was evaluated. Ki67 was expressed in all samples, with varying expression levels ranging from 1% to 95% (mean, 46%). To better address disease heterogeneity and minimize intra-group variability, the 69 lesions were categorized into eight distinct tumor subgroups based on anatomical site or tissue origin. These include conventional organ-system-based categories such as the digestive system, reproductive system, respiratory system, and nervous system. Lesions not readily classifiable into conventional organ systems, such as nasopharyngeal carcinoma and parotid gland carcinoma, were grouped under head and neck tumors. Tumors of mesenchymal origin, including stromal tumors, Ewing sarcoma, epithelioid angiosarcoma, and perivascular epithelioid cell tumors, constituted the mesenchymal-origin group. Various lymphoma subtypes were classified under hematologic tumors. Additionally, thymomas that did not fit into any of the above categories were designated as a separate thymic tumor group. The specific information on different pathological types of different groups is shown in Table 2.

**Table 2:** Pathological types and numbers across different tumor groups

Group Category	Number of lesion	Pathological Type
Digestive System	16	5 gastric adenocarcinoma; 7 colorectal cancer; 2 liver cancer; 1 cholangiocarcinoma; 1 esophageal squamous cell carcinoma
Reproductive System	15	7 cervical squamous cell carcinomas; 4 breast cancers; 2 ovarian serous carcinomas; 2 vaginal squamous cell carcinomas
Head and neck cancers	12	10 non-keratinizing squamous cell carcinomas; 1 ductal adenocarcinoma; 1 parotid gland carcinoma
Hematologic System	11	3 diffuse large B-cell lymphoma; 2 mantle cell lymphoma; 2 T-lymphoblastic lymphoma; 1 MALT lymphoma; 1 NK/T-cell lymphoma; 1 marginal B-cell lymphoma; 1 childhood follicular lymphoma
Respiratory System	6	2 lung adenocarcinomas; 2 lung squamous cell carcinomas; 2 small cell lung cancers
Mesenchymal origin	5	2 stromal tumors; 1 Ewing’s sarcoma; 1 epithelioid angiosarcoma; 1 perivascular epithelioid cell tumor
Nervous System	2	1 meningioma; 1 schwannoma
Thymic Tumor	2	2 thymic

### Exploratory of Ki67 as a predictor of visual Tracer positive

**Fig. 2:**
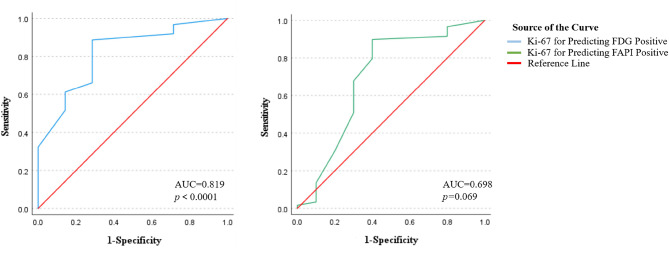
ROC curve analyses of Ki67 expression predict tracer positive

To initially elucidate the tracer-Ki67 relationship, we performed ROC curve analyses to determine if Ki67 expression could predict tracer positive. For both imaging agents, the optimal cut-off value for Ki67 was 15%. Using the 15% Ki-67 cut-off, we found that Ki67 was a powerful predictor of ^18^F-FDG positive (AUC = 0.819, 95%CI: 0.661–0.977, *p* < 0.0001). For ^68^Ga-FAPI-04, Ki67 demonstrated only a modest, non-significant predictive value (AUC = 0.698, 95%CI: 0.485–0.912, *p* = 0.069). The ROC curve analysis is shown in Fig. 2 and Table 3.

**Table 3:** Diagnostic performance of Ki67 expression for predicting visual tracer positive

Tracer	AUC (95% Confidence Interval)	*P***-value**	Optimal Ki-67 Cut-off* (%)	Sensitivity (%)	Specificity (%)
^18^F-FDG	0.819 (0.661–0.977)	＜0.0001	15	88.7	71.4
^68^Ga-FAPI-04	0.698 (0.485–0.912)	0.069	15	89.8	60.0

*The optimal Ki67 cut-off value was determined by maximize of the Youden index (Sensitivity + Specificity − 1)

Note: Tracer positive was determined by visual assessment of PET/CT images

### ^68^Ga-FAPI-04 vs.^18^FDG PET imaging

For the purpose of acquiring a preliminary understanding of the fundamental characteristics of the uptake of two imaging agents, we investigated whether the SUVmax and TBR differed across various tumor groups. After confirming non-normal data distribution, the Kruskal-Wallis test was employed for comparisons. The analysis revealed no statistically significant differences in SUVmax of ^18^F-FDG across different tumor groups (*p* = 0.260), whereas SUVmax of ^68^Ga-FAPI-04 exhibited significant group heterogeneity (*p* = 0.002). Similarly, while TBR of ^18^F-FDG demonstrated no significant variation across tumor groups (*p* = 0.072), TBR of ^68^Ga-FAPI-04 showed statistically significant differences (*p* = 0.004). The mean SUVmax and TBR values for each tumor group are presented in Table 4.

**Table 4:** The mean SUVmax and TBR in lesions of different tumor groups

Different groups	Number of lesion	SUVmax				TBR			
		^18^F-FDG	*P* value	^68^Ga-FAPI	*P* value	^18^F-FDG	*P* value	^68^Ga-FAPI	*P* value
Digestive System	16	10.04±11.43	0.260	10.16±4.44	0.002	5.25±5.00	0.072	9.17±5.29	0.004
Reproductive System	15	12.65±7.29		14.22±7.22		8.89±9.31		12.18±5.48	
Head and neck cancers	12	12.88±6.31		10.92±4.33		7.05±3.89		10.15±4.66	
Hematologic System	11	19.99±17.61		6.44±3.26		15.27±14.49		6.30±3.57	
Respiratory System	6	6.63±3.51		5.95±2.95		3.88±1.85		5.12±2.23	
Mesenchymal origin	5	8.9±3.16		10.54±2.54		4.91±2.33		8.81±1.19	
Nervous System	2	9.1±2.97		4±2.55		4.19±1.62		2.71±1.00	
Thymic Tumor	2	7.75±5.3		3.75±2.19		3.83±1.65		2.96±1.59	

Additionally, we performed a correlation analysis of the same parameters between ^18^F-FDG and ^68^Ga-FAPI-04. Across all lesions, the results demonstrated a moderate positive correlation in SUVmax between the two tracers (rho = 0.430, *p* < 0.001), and a similarly moderate correlation in TBR (rho = 0.391, *p* < 0.001). Subsequently, we stratified the lesions based on Ki67 expression levels, adopting a cut-off of 15% from previous analysis. Correlation analyses between the two tracers were then conducted within these subgroups. In the 12 lesions of Ki67≤15%, both SUVmax and TBR showed significant correlations (SUVmax: rho = 0.625, *p* = 0.033; TBR: rho = 0.701, *p* = 0.014). In the Ki67＞15% group of 57 lesions, a correlation was also observed between the SUVmax values of the two imaging agents, albeit weakly (rho = 0.329, *p* = 0.013). Furthermore, the correlation for TBR was even weaker (rho = 0.295, *p* = 0.026). The correlation between the two PET tracers is shown in the Table 5.

**Table 5:** Correlation of parameters of two agents across different Ki67 expression

Ki67 expression	Number of lesion	SUVmax’-SUVmax’’		TBR’-TBR’’	
		rho	*P* value	rho	*P* value
≦15	12	0.625	0.033	0.701	0.014
> 15	57	0.329	0.013	0.295	0.026
Total	69	0.430	＜0.001	0.391	＜0.001

Note: SUVmax’ means SUVmax of ^18^F-FDG; SUVmax’’ means SUVmax of ^68^Ga-FAPI-04; TBR’ means TBR of ^18^F-FDG; TBR’’ means TBR of ^68^Ga-FAPI-04

### Correlation of parameters and Ki67 immunohistochemistry

**Fig. 3:**
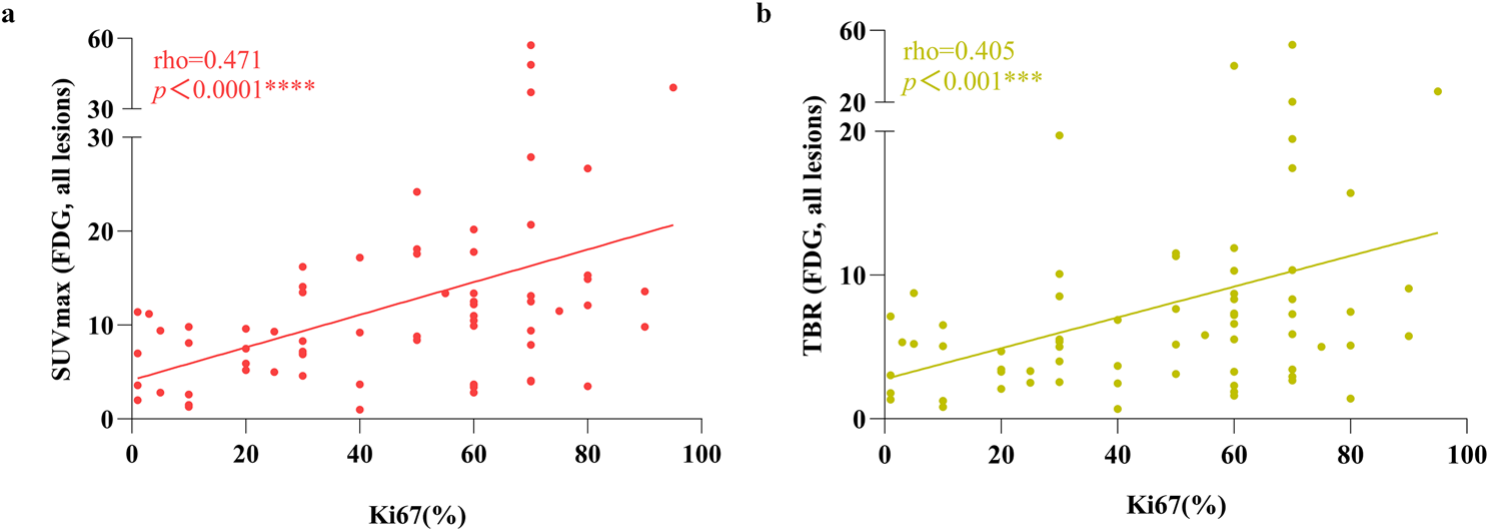
Correlation between ^18^F-FDG PET/CT parameters and Ki67 expression

**Fig. 4:**
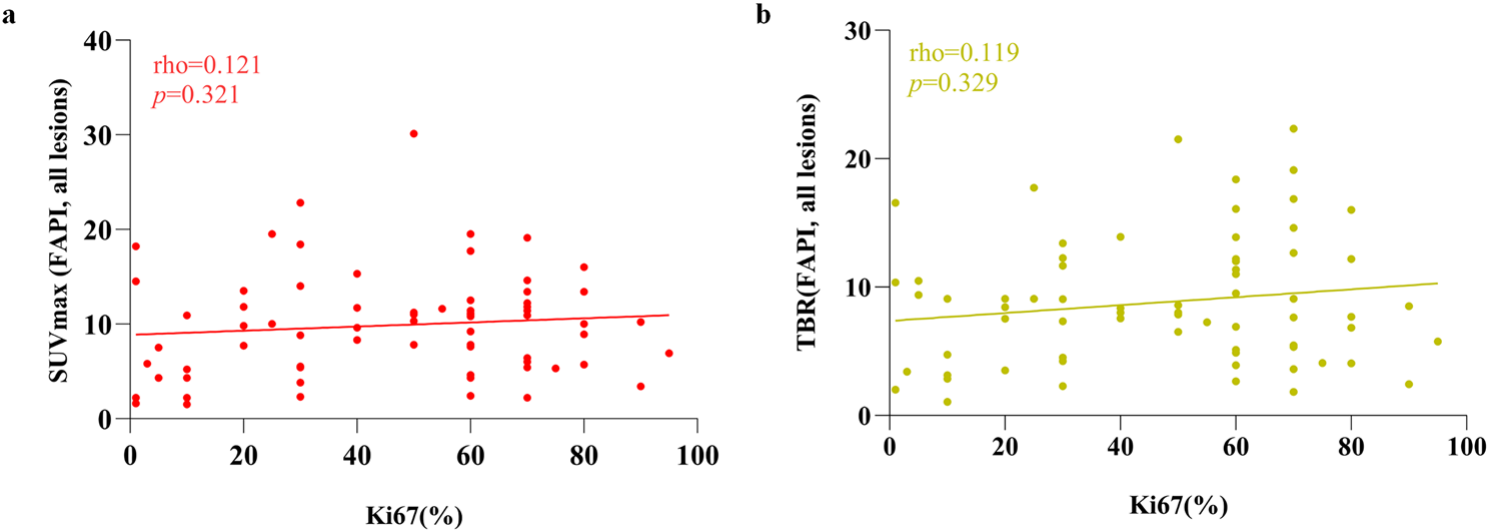
Correlation between ^68^Ga-FAPI-04 PET/CT parameters and Ki67 expression

In general, both SUVmax and TBR of ^18^F-FDG exhibited a correlation with Ki67 (Fig. 3), with demonstrating a moderate correlation (SUVmax: rho = 0.471, *p* < 0.0001 and TBR: rho = 0.405, *p* < 0.001, respectively). Based on the analysis of all lesions, neither SUVmax nor TBR of ^68^Ga-FAPI-04 showed any correlation with Ki67 (Fig. 4, SUVmax: rho = 0.121, *p* = 0.321 and TBR: rho = 0.119, *p* = 0.329, respectively).

We used the Ki67 cut-off value of 15% obtained from the previous ROC curve analysis as the stratification criterion to further stratify the lesion tissue and explore possible threshold effects. In the Ki67≤15% subgroup, no significant correlations were observed between Ki67 expression and either ^18^F-FDG parameters (SUVmax: *p* = 0.332; TBR: *p* = 0.383) or ^68^Ga-FAPI-04 parameters (SUVmax: *p* = 0.456; TBR: *p* = 0.486). However, in lesions with Ki67 > 15%, ^18^F-FDG maintained significant moderate correlations with Ki67 expression (SUVmax: rho = 0.346, *p* = 0.008; TBR: rho = 0.327, *p* = 0.013). Notably, even in this subgroup, ^68^Ga-FAPI-04 parameters continued to show no significant correlation with Ki67 expression (SUVmax: *p* = 0.349; TBR: *p* = 0.726). We have presented the above data in the form of a Table 6.

**Table 6:** Correlation of parameters of two tracers and different Ki67 subgroups

Ki67 expression	Number of lesion	SUVmax-Ki67				TBR-Ki67			
		rho ^(18^F-FDG^)^	*P* value	rho (^68^Ga-FAPI-04)	*P* value	rho ^(18^F-FDG^)^	*P* value	rho (^68^Ga-FAPI-04)	*P* value
≦15	12	−0.304	0.332	−0.234	0.456	−0.274	0.383	−0.219	0.486
> 15	57	0.346	0.008	−0.126	0.349	0.327	0.013	−0.048	0.726
Total	69	0.471	< 0.0001	0.121	0.321	0.406	< 0.001	0.120	0.326

Furthermore, we conducted a subgroup analysis for tumor groups with more than 10 lesions. In the digestive system, there was no correlation observed in SUVmax and TBR for either ^18^F-FDG or ^68^Ga-FAPI-04 with Ki67 expression (SUVmax: *p* = 0.908 and 0.812, respectively. TBR: *p* = 0.967 and 0.706, respectively). In the reproductive system, only SUVmax of ^18^F-FDG demonstrated high correlation with Ki67 (rho = 0.676, *p* = 0.007). In head and neck tumors, the SUVmax and TBR of ^18^F-FDG exhibited high correlation with Ki67 (SUVmax: rho = 0.627, *p* = 0.033 and TBR: rho = 0.585, *p* = 0.049, respectively). In hematological tumors, Ki67 had very strong correlation with SUVmax of ^18^F-FDG (rho = 0.827, *p* = 0.003), while its TBR showed high correlation (rho = 0.659, *p* = 0.031). In each tumor group, the correlation between Ki67 expression and ^68^Ga-FAPI-04 (SUVmax and TBR) was not statistically significant. The detailed correlation results are presented in Table 7.

**Table 7:** Correlation of parameters of two agents and Ki67 in different lesion-groups

Different lesion-group	Number of lesion	SUVmax-Ki67				TBR-Ki67			
		rho ^(18^F-FDG^)^	*P* value	rho (^68^Ga-FAPI-04)	*P* value	rho ^(18^F-FDG^)^	*P* value	rho (^68^Ga-FAPI-04)	*P* value
Digestive System	16	−0.03	0.908	0.064	0.812	−0.012	0.967	0.102	0.706
Reproductive System	15	0.676	0.007	0.121	0.666	0.503	0.058	0.213	0.443
Head and neck cancers	12	0.627	0.033	0.095	0.769	0.585	0.049	0.222	0.485
Hematologic System	11	0.827	0.003	0.383	0.244	0.659	0.031	0.178	0.600

## Discussion

This lesion-based study evaluated the relationships between Ki67 expression and the semi-quantitative PET parameters of ^18^F-FDG and ^68^Ga-FAPI-04, acrossing a broad spectrum of malignancies. Our multifaceted analysis reveals that ^18^F-FDG serves as a valuable, non-invasive tool for tumor proliferative activity, whereas ^68^Ga-FAPI-04 may provide complementary information that is independent of cellular proliferation, reflecting the tumor stromal microenvironment.

**Fig. 5:**
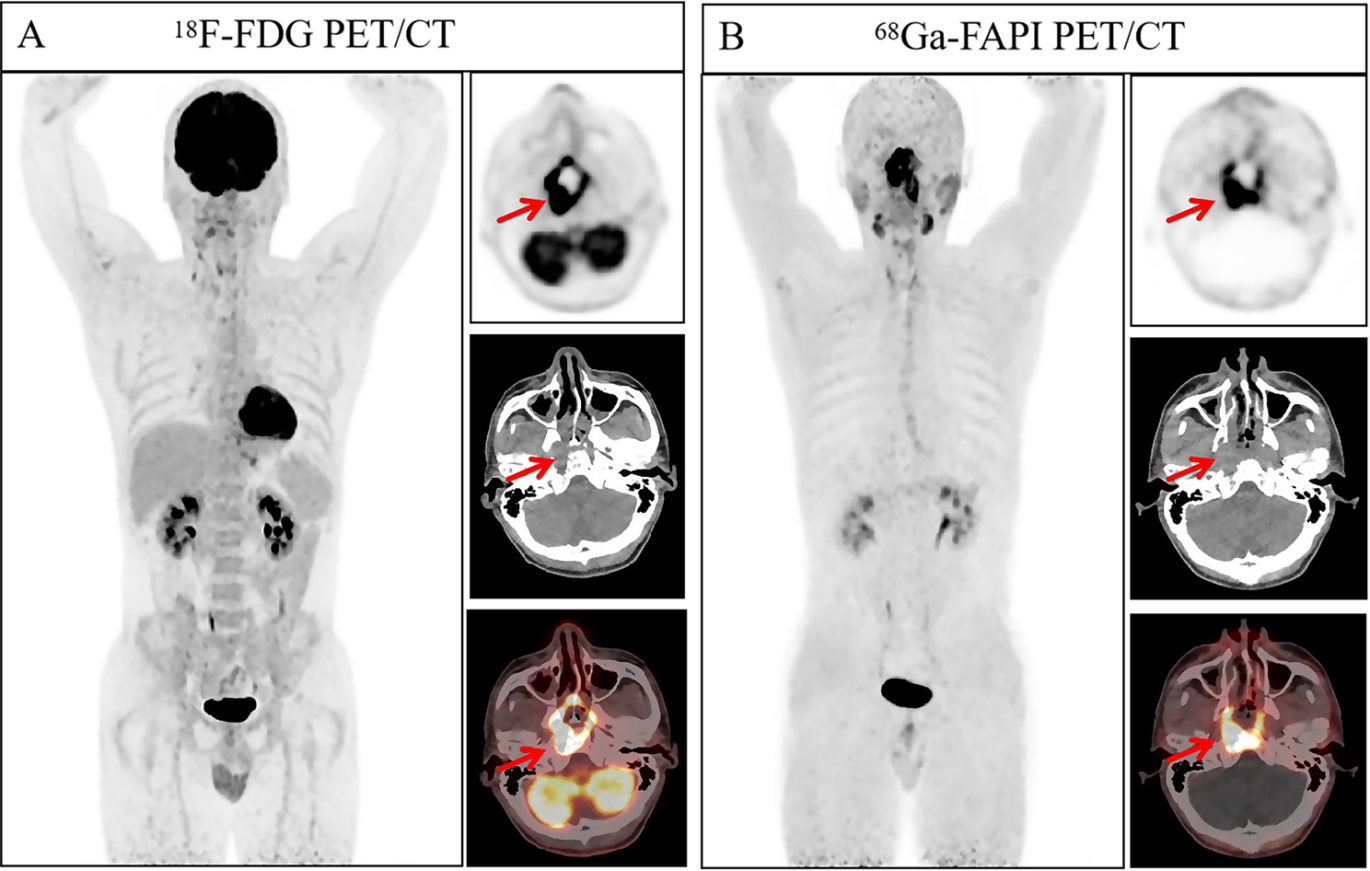
A 37-year-old man, showing thickening of the nasopharyngeal posterior wall with increased radiotracer uptake (indicated by the arrows. ^18^F-FDG-SUVmax 26.70, ^18^F-FDG-TBR 15.71, ^68^Ga-FAPI-04-SUVmax 13.40, and ^68^Ga-FAPI-04-TBR 12.18). Pathological examination of the nasopharyngeal posterior wall neoplasm was diagnosed as non-keratinizing squamous cell carcinoma (undifferentiated type). Immunohistochemical findings: Ki67 (+, 80%)

**Fig. 6:**
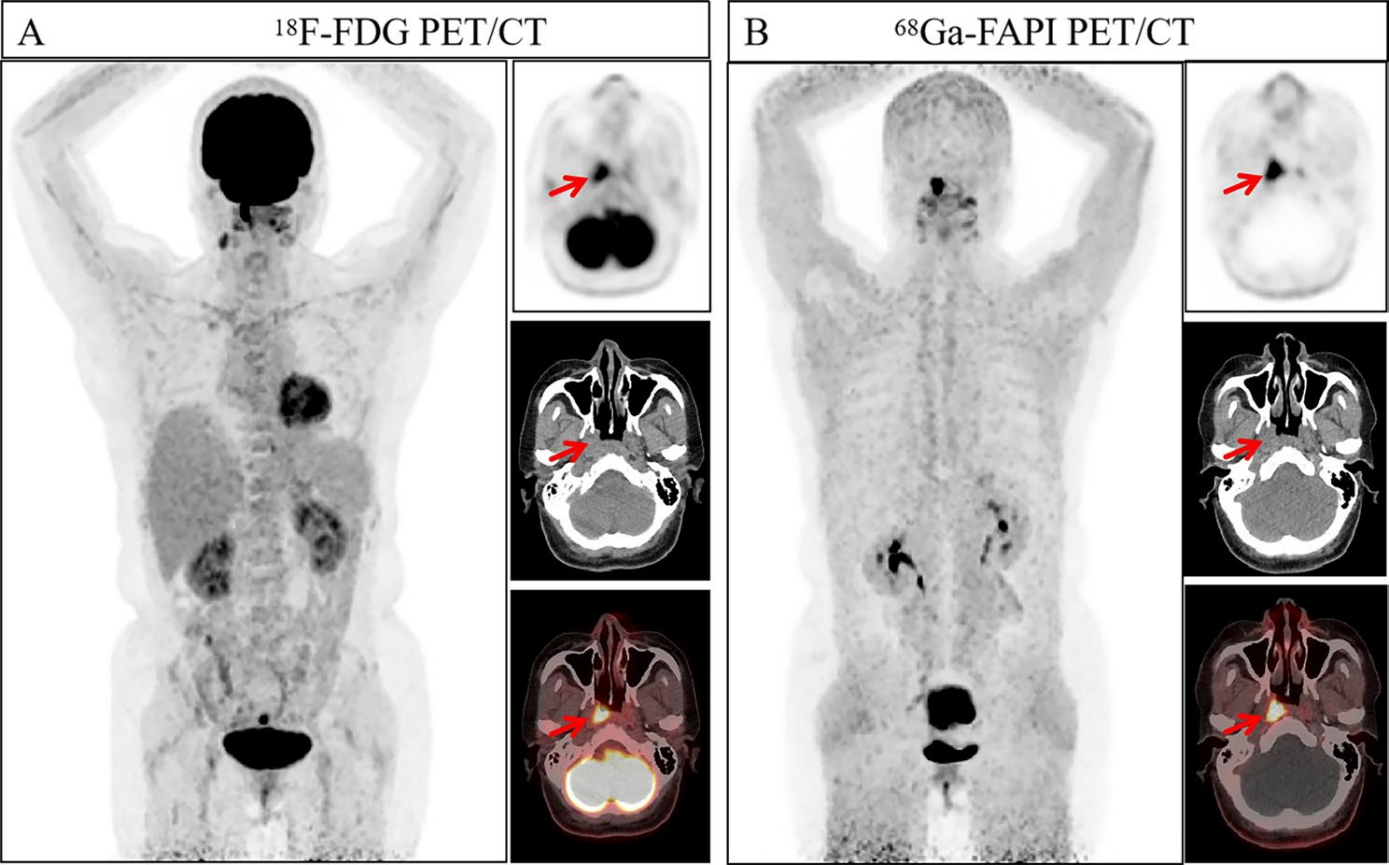
A 53-year-old woman, demonstrating thickening of the nasopharyngeal posterior wall. Pathological analysis of the nasopharyngeal posterior wall neoplasm suggested a malignant tumor, further correlation with immunohistochemical and in situ hybridization results confirmed the diagnosis of non-keratinizing squamous cell carcinoma (undifferentiated type). Immunohistochemical findings: Ki67 (+, 40%). Regarding radiotracer uptake in imaging examinations, increased uptake was observed for both tracers, with higher uptake of ^68^Ga-FAPI-04 PET/CT compared to ^18^F-FDG PET/CT (indicated by the arrows. ^18^F-FDG-SUVmax 9.20, ^18^F-FDG-TBR 3.68, ^68^Ga-FAPI-04-SUVmax 11.70, and ^68^Ga-FAPI-04-TBR 8.36)

**Fig. 7:**
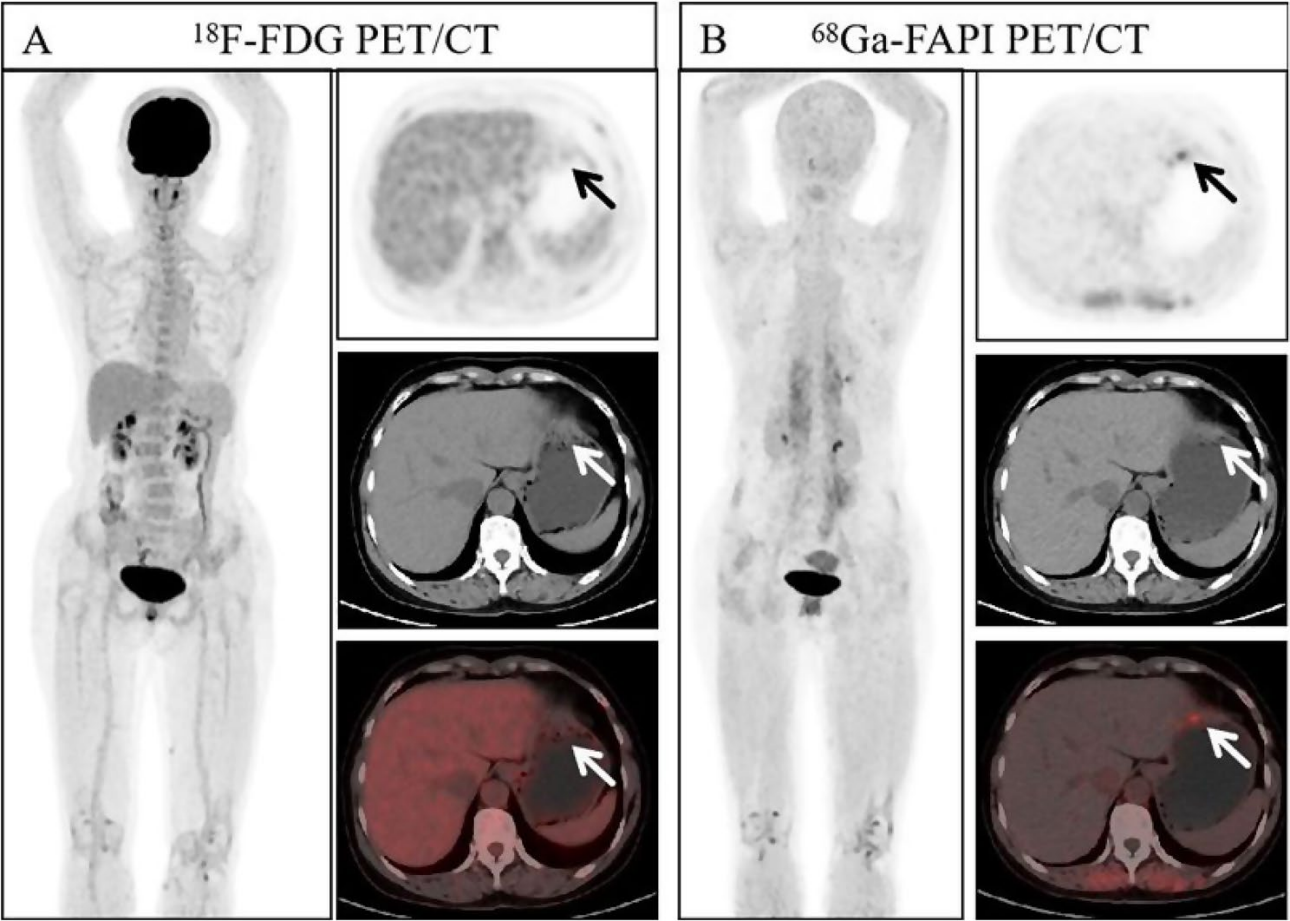
A 54 year old woman presented with gastric discomfort, resulting in slight thickening of the soft tissue in the central part of the stomach (as indicated by the arrows), slightly increased uptake of ^18^F-FDG PET/CT (SUVmax 2.60, TBR 1.24), and increased uptake of ^68^Ga-FAPI-04 PET/CT (SUVmax 4.30, TBR 2.87). Subsequently, she underwent gastroscopy biopsy, and the tissue sent for examination in the middle of the stomach showed atrophy with erosive and regenerative changes. A large amount of lymphoid tissue hyperplasia was observed in the gastric mucosa. Combined with immunohistochemical results and clinical observations, it was considered that mucosa associated lymphoid tissue was associated with B-cell lymphoma in the lymph node outer margin. Immunohistochemistry: Ki67 (+, 10%)

**Fig. 8:**
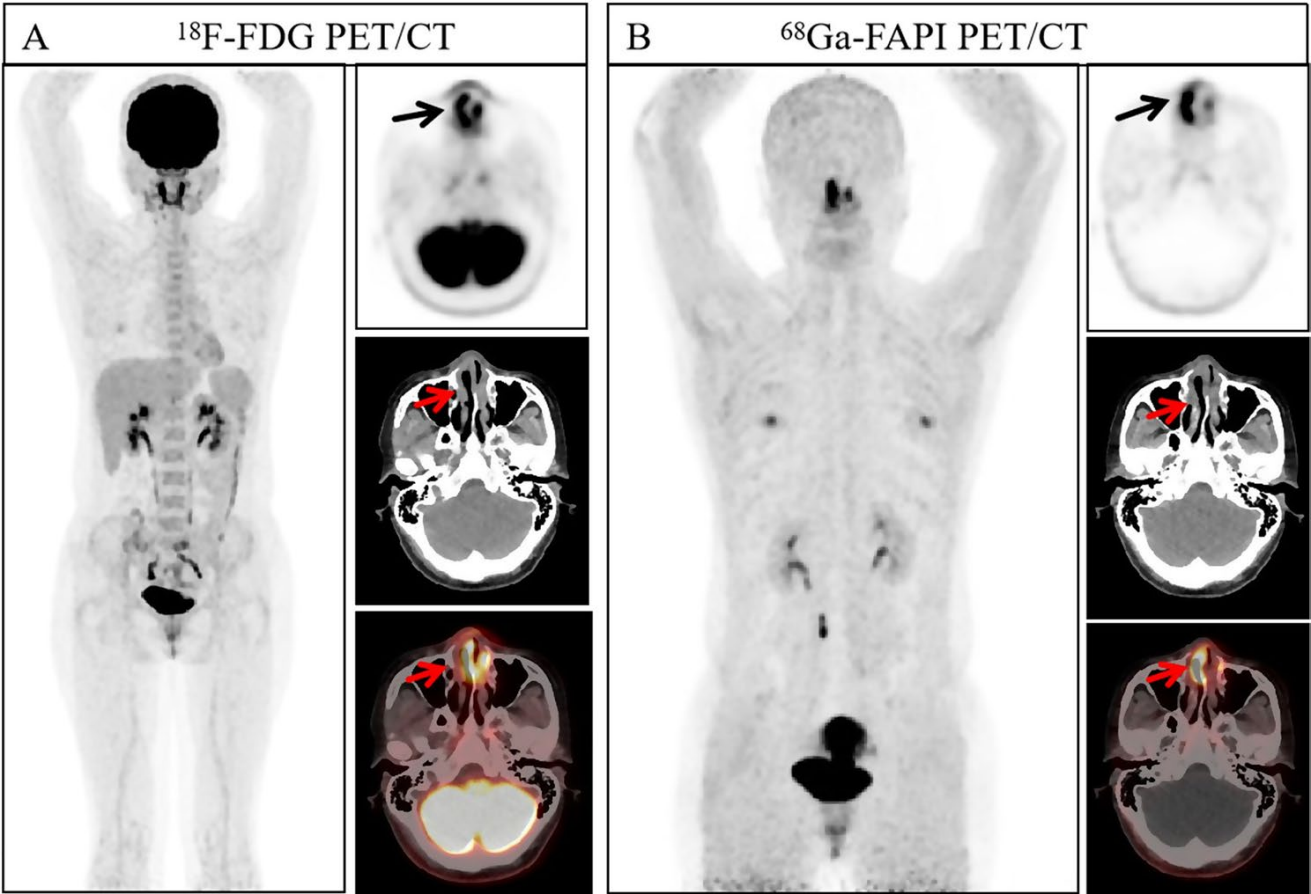
A 31 year old woman with a mass in her right nasal cavity was diagnosed with extranodal NK/T-cell lymphoma, nasal type, through pathological biopsy. Immunohistochemistry: Ki67 (+, 60%). In both imaging agents, there was an increase in uptake. (as indicated by the arrow, ^18^F-FDG-SUVmax 9.90, ^18^F-FDG-TBR 6.60, ^68^Ga-FAPI-04-SUVmax 11.00, and ^68^Ga-FAPI-04-TBR 11.00)

**Fig. 9:**
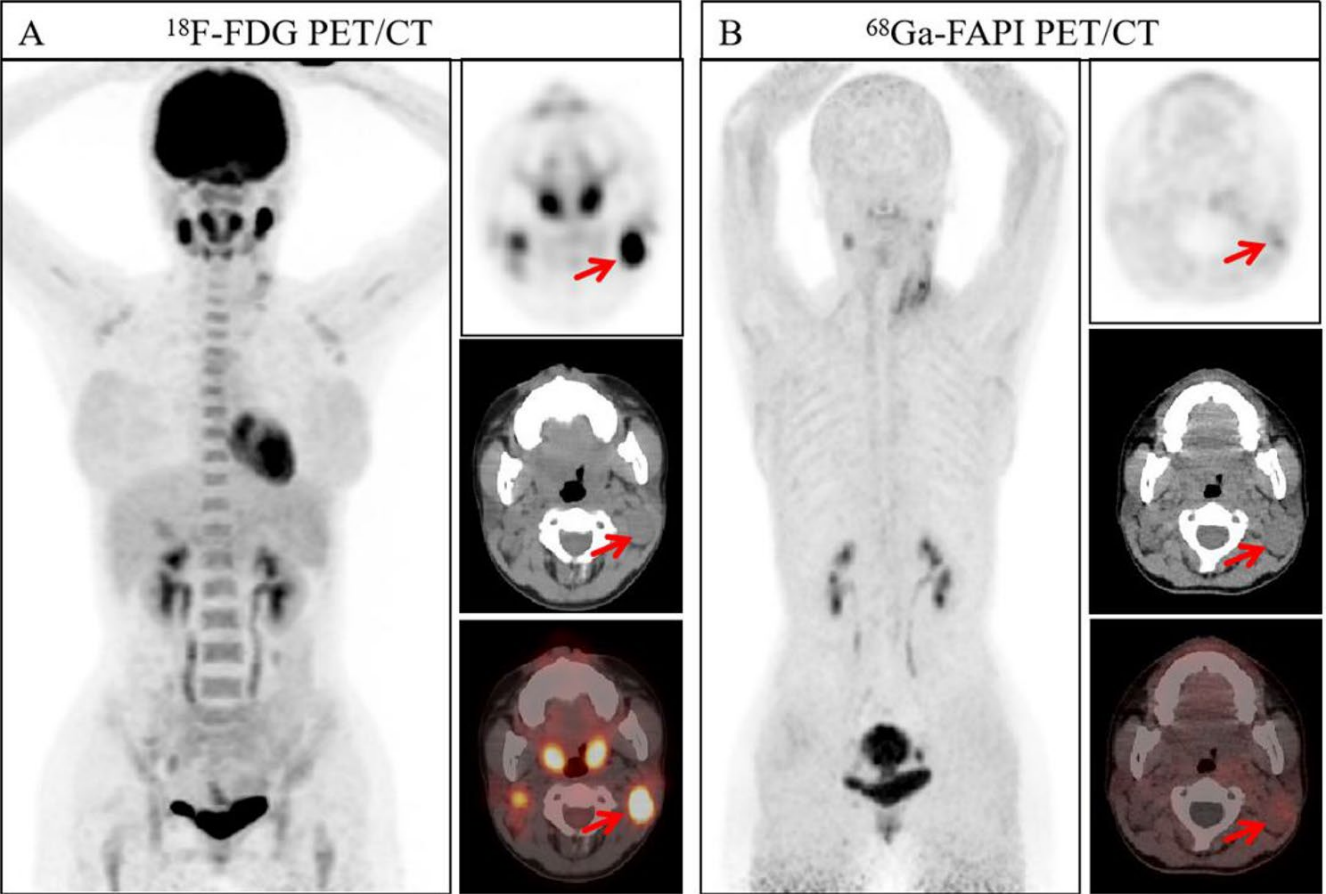
A 22-year-old woman presented with enlarged left lymph nodes and underwent pathological biopsy, which revealed childhood follicular lymphoma. Immunohistochemistry: Ki67 (+, 90%). The lesion showed a significant increase in uptake on ^18^F-FDG PET/CT (arrows, SUVmax 13.6, TBR 9.07), while the degree of the uptake of ^68^Ga-FAPI-04 PET/CT was not high (arrows, SUVmax 3.4, TBR 2.43)

Our primary results demonstrate that, in the overall analysis, ^18^F-FDG uptake shows a moderate correlation with Ki67, while no significant correlation was observed between ^68^Ga-FAPI-04 and Ki67. This reaffirms that ^18^F-FDG and Ki67 share the core biological process of cell proliferation. The correlation between ^18^F-FDG and Ki67 was particularly pronounced in head and neck tumors (Figs. 5 and 6) and hematological malignancies (Figs. 7, 8 and 9), consistent with findings by Minn et al. [15] in head and neck tumors and aligning with the highly aggressive, glycolytically active biology of lymphomas with minimal stromal interference [16]. Furthermore, the optimal Ki67 cut-off value of 15% determined by our ROC curve analysis is consistent with previously established classifications distinguishing low from moderate-to-high proliferative tumors [17], further validating the clinical potential of ^18^F-FDG for predicting Ki67 expression in malignancies with moderate to high proliferation. Our results are largely congruent with numerous prior studies that confirmed positive correlations between ^18^F-FDG PET/CT parameters and Ki67 expression in neuroendocrine tumors [18, 19], meningiomas [20], and ovarian cancers [21].

Regarding ^68^Ga-FAPI-04, its lack of correlation with Ki67 across all stratified analyses, consistent with the ROC curve results, indicates its limited capability for assessing proliferative activity. The result aligns with a previous study by Ruan et al. on gliomas [11]. However, this relationship appears to be cancer-type-specific. For instance, a recent head-to-head study by Liang et al. reported a linear correlation between ^18^F-FAPI-04 SUVmax and Ki67 in liver cancer [10]. Besides, a previous study by Kreppel et al. on hepatic metastases of neuroendocrine tumors also found no statistically significant correlation between SUVmax of ^68^Ga-DATA^5^ m.SA.FAPi and Ki67 expression [19]. This is primarily attributable to the specific targeting mechanism of ^68^Ga-FAPI-04, which primarily binds to CAFs in the tumor stroma rather than acting directly on tumor cells themselves.

A noteworthy observation was that the correlation between the parameters of ^18^F-FDG and ^68^Ga-FAPI-04 was stronger in the Ki67≤15% low-proliferation subgroup but weakened in the Ki67 > 15% high-proliferation subgroup. We hypothesize that in low-proliferation tumors, metabolic activity and stromal activation might be driven by a common upstream signal. In contrast, in highly proliferative tumors, malignant cell proliferation and the stromal response may progressively diverge onto distinct regulatory pathways, leading to the dissociation between ^18^F-FDG and ^68^Ga-FAPI uptake. Additionally, although the “Warburg effect” is a common feature of malignancies, and certain specific tumor types, such as hepatocellular carcinoma, renal cell carcinoma, are typically characterized by low ^18^F-FDG uptake [22]. No significant differences in ^18^F-FDG uptake were observed among different tumor groups in our specific cohort. This might be related to the insufficient number of typical FDG-low expressing tumors in our sample. In contrast, ^68^Ga-FAPI-04 uptake showed significant inter-tumor heterogeneity, underscoring that stromal FAP expression is highly dependent on specific tumor types and their unique microenvironments, consistent with the known biological diversity of CAFs [9].

Our findings support and reaffirm relevant tumor biology theories. The correlation between ^18^F-FDG and Ki67 reinforces the theoretical link between tumor cell proliferation and glycolytic metabolism. Conversely, although multiple studies have shown that ^68^Ga-FAPI is superior to ^18^F-FDG in lesion diagnosis [23–27], the lack of correlation between ^68^Ga-FAPI-04 and Ki67 corroborates the theory that stromal activation is a process relatively parallel yet independent of tumor cell proliferation. Essentially, FAP is a marker for activated stromal CAFs, facilitating tumor microenvironment remodeling, whereas Ki67 is a proliferation marker of tumor cells, reflecting cell division activity. Furthermore, while ^18^F-FDG PET shows promise as a non-invasive indicator of proliferative activity, its primary clinical utility, akin to the established role of FDG PET in neuroendocrine tumors, likely lies not in replacing biopsy but in profiling intra- and inter-lesional heterogeneity. It could guide biopsies to the most FDG-avid sites and identify aggressive subclones within metastatic disease, especially in lesions inaccessible or unsuitable for repeated biopsy. In the era of precision medicine, appropriate imaging agents should be selected based on clinical needs or used in combination to obtain more comprehensive tumor biological information.

This study has some limitations. Firstly, the included patients come from a single institution with a small sample size (*n* = 69). The limited number of lesions within each tumor groups and the inclusion of various pathological subtypes within them preclude drawing definitive conclusions for any specific cancer type from our data. These subgroup results should be considered preliminary, highlighting the need for validation in larger, more homogeneous patient cohorts. Secondly, we only analyzed the SUVmax and TBR of two imaging agents. Although Ki67 serves as a marker of tumor proliferation, it is often determined based on single-point biopsy, whereas PET imaging covers almost the entire lesion. In such cases, the biopsy area may not accurately represent the metabolic or stromal status of the entire lesion. To ensure the rigor of our study, we strive to align the biopsy site as closely as possible with the ROI area outlined on the PET scan. These limitations, along with the findings of our study, underscore the significance of larger sample sizes and subgroup analysis in future research to explore the correlation between Ki67 and more PET/CT parameters. Lastly, this study did not evaluate the comparative utility of ^68^Ga-FAPI-04 and ^18^F-FDG for clinical staging or restaging of the specific tumor types included. Investigating their impact on clinical management remains an important area for future studies.

## Conclusion

In summary, our findings indicate that ^18^F-FDG may serve as a potential non-invasive preliminary imaging modality for assessing Ki67 across all lesions, particularly in medium-to-high grade malignancies. While ^68^Ga-FAPI-04 parameters are unsuitable for non-invasive Ki67 evaluation, they may act as a complementary indicator to reflect tumor microenvironment characteristics. The subgroup-specific correlations require further validation in larger and dedicated studies.

## Data Availability

The datasets generated during and/or analyzed during the current study are available from the corresponding author upon reasonable request.

## Abbreviations

^18^F-FDG: Fluorine-18-fluorodeoxyglucose
^68^Ga-FAPI: Gallium-68-fibroblast activation protein inhibitor
PET: Positron emission tomography
CT: Computed tomography
SUVmax: Maximum standardized uptake values
TBR: Tumor-to-background ratio
CAFs: Cancer-associated fibroblasts
SUVmean: Mean standardized uptake value
ROC: Receiver operating characteristic
AUC: Area under the receiver operating characteristic curve
